# Fairchild, Bro. & Foster

**Published:** 1885-10-20

**Authors:** 


FAIRCHILD BRO. & FOSTER.—We invite attention to the change of advertisement of this reliable and excellent House, as found on colored insert in this journal. The process suggested more closely approximates normal human milk than any other heretofore devised. We have found good results with the Peptogenic Milk Powders in practice.

